# Immobilization of Polyoxometalates on Tailored Polymeric Surfaces

**DOI:** 10.3390/nano8030142

**Published:** 2018-03-02

**Authors:** Saioa Aguado-Ureta, Juan Rodríguez-Hernández, Adolfo del Campo, Leyre Perez-Álvarez, Leire Ruiz-Rubio, José Luis Vilas, Beñat Artetxe, Santiago Reinoso, Juan M. Gutiérrez-Zorrilla

**Affiliations:** 1 Departamento de Química Inorgánica, Facultad de Ciencia y Tecnología, Universidad del País Vasco UPV/EHU, P.O. Box 644, 48080 Bilbao, Spain; saioa.aguado@ehu.eus (S.A.-U.); benat.artetxe@ehu.eus (B.A.); santiago.reinoso@unavarra.es (S.R.); 2 Departamento de Química y Propiedades de Materiales Poliméricos, Instituto de Ciencia y Tecnología de Polímeros (ICTP-CSIC), C/Juan de la Cierva 3, 28006 Madrid, Spain; jrodriguez@ictp.csic.es; 3 Departamento de Electrocerámica, Instituto de Cerámica y Vidrio (ICV-CSIC), C/Kelsen 5, 28049 Madrid, Spain; adelcampo@icv.csic.es; 4 Grupo de Química Macromolecular (LABQUIMAC), Departamento de Química Física, Facultad de Ciencia y Tecnología, Universidad del País Vasco UPV/EHU, P.O. Box 644, 48080 Bilbao, Spain; leyre.perez@ehu.eus (L.P.-Á.); leire.ruiz@ehu.eus (L.R.-R.); 5 BCMaterials, Basque Center for Materials, Applications and Nanostructures, UPV/EHU Science Park, 48940 Leioa, Spain

**Keywords:** nanocluster, polyoxometalates, functionalized polymer surfaces, interfacial migration

## Abstract

Herein we describe the preparation of hybrid polymer–inorganic interfaces by the immobilization of polyoxometalate nanoclusters on functionalized polymer surfaces. The polymeric surfaces were made of polystyrene-*b*-poly(acrylic acid)/polystyrene (PS-*b*-PAA/PS) blends by spin coating on a silicon wafer. The functionalization of the polymer film was obtained by interfacial migration of the amphiphilic block copolymer toward the interface upon water vapor annealing. The carboxylic acid functional groups contained in the PAA block were then employed to anchor the [Ln^III^(α-SiW_11_O_39_)]^5−^ polyoxometalates (Ln: Ce, Er). This purpose was achieved by immersing the films in aqueous solutions of the in situ-formed inorganic nanoclusters. X-ray photoelectron and confocal Raman spectroscopies, together with atomic force microscopy, confirmed the immobilization of the inorganic species at the interface.

## 1. Introduction

Organic–inorganic composites represent one of the current hot topics in materials science due to the possibility of combining the specific characteristics of two different components in a single material to obtain unusual properties that may result in novel applications. The association of inorganic and organic species in hybrid composites has made available a vast scientific area around the development of multifunctional materials.

In particular, polyoxometalates (POMs) are widely recognized as one of the most interesting types of inorganic components that are suitable for being incorporated into multifunctional materials [1,2,3,4]. This well-known class of nanosized anionic nanoclusters with metal-oxo frameworks offers outstanding features, such as (i) possessing high solution, thermal, and oxidative stability; (ii) including species with a wide range of well-defined sizes and shapes, often with highly symmetric topologies; (iii) having interesting electronic properties (e.g., storage of multiple electrons/protons without substantial skeletal modifications) that can be tuned to a great extent by systematic compositional variations on the POM frameworks; (iv) acting as multidentate inorganic ligands to incorporate either organic moieties with additional functionalities or extra d- or f-block metallic centers [5,6,7,8,9,10]. These features have allowed POMs to find potential applications in diverse fields (e.g., catalysis, magnetism, biomedicine, spintronics, molecular recognition, optics, conductivity, ion exchange) with implications in current issues of interest related to technology, health, energy, and the environment [11,12,13,14,15]. As inorganic components, POMs have, for example, been combined with amphiphilic molecules or cationic surfactants to construct several discrete architectures (micelles, capsules, vesicles, cones), fibers and wires, or highly ordered bidimensional arrays (self-assembled monolayers, Langmuir and Langmuir–Blodgett films, layer-by-layer structures) [16,17,18,19,20,21,22,23]. They have also been incorporated into several types of organic materials, such as carbon nanotubes, graphene, metal–organic frameworks, and polymeric matrices (either by adsorption or co-polymerization when derivatized with suitable functionalities) [24,25,26,27,28,29,30,31,32,33].

Immobilization of POMs on solid surfaces represents a key step toward processing these nanoclusters in functional devices [34]. For example, the anchorage of a given POM to a solid substrate can lead to the combination of its catalytic activity in homogeneous phase with the ease of recovery and recycling shown by heterogeneous catalysts, which represents a clear demand for industrial purposes [35,36]. Different solid substrates have been employed as POM supports, such as oxides (alumina, silica), metals (silicon, gold), and HOPG [37,38,39,40,41,42,43]. Since POMs are negatively charged, their immobilization usually relies on electrostatic interactions, which might result in partial leaching of these clusters. Surfaces are usually functionalized with positively charged residues and/or H-donor groups to enhance the electrostatic interactions and/or to generate a reinforcing network of hydrogen bonds, but this does not ensure that leaching is completely avoided. Different strategies based on the covalent linkage of POMs have been applied to overcome this [44,45,46], such as using organically modified POMs and substrates bearing complementary functionalities for covalent bond formation (e.g., amino and carboxylic groups) [47,48,49] or grafting N-donor groups on the solid surface to coordinate transition-metal substituted POMs with terminal aqua ligands [50,51]. However, the former approach limits the catalog of suitable POMs mainly to lacunary derivatives of the Keggin and Wells–Dawson anions, whereas the strength of the POM-surface linkage might still be questionable in the latter approach due to the monodentate character of the coordinating groups selected so far.

Nevertheless, the coordinative strategy could be improved by taking advantage of chelation, which requires that the surfaces are functionalized with coordinating groups of polydentate character and that the POMs contain exposed metal centers with at least two terminal and labile water molecules in their coordination spheres. Herein we describe the preparation of functionalized films composed of polystyrene-*b*-poly(acrylic acid)/polystyrene (PS-*b*-PAA/PS) blends. As will be depicted, the surface segregation of the poly(acrylic acid) block allows the immobilization of POMs via their flexible polystyrene branches terminated with O-donor polycarboxylic residues. The polymeric surfaces were made by spin coating on a silicon wafer and functionalized via interfacial migration of the amphiphilic block copolymer towards the interface upon water vapor annealing, which represents a well-established method for designing functional polymeric films [52,53]. The nanoclusters selected as models to test the POM anchoring capability of these films were of the type [Ln^III^(α-SiW_11_O_39_)]^5−^ (Ln: Ce, Er). They are composed of a Keggin monolacunary subunit acting as a tetradentate ligand toward an oxophilic 4f ion, whose coordination sphere in aqueous solution is completed by at least four water molecules that are labile, as indicated by the monodimensional assemblies in which they tend to crystallize [54,55,56,57,58]. To our knowledge, only a few examples of hybrid film composites involving POMs and diblock copolymers have been described in the literature up to now. These include polythiophene-based hybrid materials for photovoltaic solar cells that contain covalently linked POMs [59,60,61] and systems in which the formation of aggregates in solution are employed to direct the self-assembly of highly ordered films with inverse hexagonal topology [62].

## 2. Materials and Methods

The K_8_[α-SiW_11_O_39_]·13H_2_O precursor was synthesized according to literature procedures and identified by infrared spectroscopy [63]. All other chemicals were obtained from commercial sources and used without further purification. Fourier transform infrared (FT-IR) spectra were recorded on KBr pellets using a Shimadzu FTIR-8400S spectrophotometer (Shimadzu, Kyoto, Japan).

X-ray photoelectron spectroscopy (XPS) measurements were performed in a SPECS system (SPECS Surface Nano Analysis, Berlin, Germany) equipped with a Phoibos 150 1D-DLD analyzer (Berlin, Germany) and Al Kα monochromatic radiation source (1486.6 eV). Initial analysis of the elements present in the samples was conducted applying the following conditions: step energy 1 eV, dwell time 0.1 s, pass energy 40 eV. The elements of interest were further analyzed in detail with an electron exit angle of 90°. The conditions used for these detailed analyses were as follows: step energy 0.1 eV, dwell time 0.1 s, and pass energy 20 eV.

High-resolution images of Raman depth profile mapping were obtained using a WITec ALPHA 300RA confocal Raman microscope (WITec, Ulm, Germany) with a Nd:YAG laser excitation at 532 nm and a 100× objective (Numerical Aperture = 0.9). The optical resolution of the confocal microscope is limited to 200 nm in the lateral direction and to 500 nm in the vertical axis. Raman spectral resolution of the system is down to 0.02 cm^−1^. The samples were mounted in a piezo-driven scan platform having a positioning accuracy of 4 nm in the lateral direction and 0.5 nm in the vertical. The piezoelectric scanning table allows three-dimensional displacements in steps of 3 nm, leading to a very high spatial resolution. The microscope base is also equipped with a vibration isolation system active in the range 0.7–1000 Hz. In order to minimize the heating effect over the sample, a laser power of 5 mW was used with an integration time of 2 s. Each Raman image consists of ~1500 simple spectra with an integration time of 2 s, so that the total measurement time for each image was ca. 50 min. The acquired spectra were processed and analyzed using the WITec Project 2.02 software (Ulm, Germany). This software allows specific, sensitive, and non-intrusive analysis of the collected spectra that is immune to interferences, and provides a method for characterizing the chemical properties of heterogeneous samples with great resolution and rapid data collection.

Atomic force microscopy (AFM) measurements were carried out using a Nanotec Electrónica equipment (Nanotec Electrónica, Madrid, Spain). Images were recorded under room atmosphere in tapping mode and they were processed with the WSxM 5.0 software (Madrid, Spain) [64].

Synthesis of the copolymer polystyrene-*b*-poly(acrylic acid) (PS_19_-*b*-PAA_10_) was made in three steps:

Synthesis of the Polystyrene (PS-Br) Macroinitiator by atom transfer radical polymerization (ATRP). The polystyrene was synthesized by atom transfer radical polymerization (ATRP). The polymerization was performed in a Schlenk flask. ATRP was carried out using the following stoichiometry: [M]/[I]/[CuBr]/[L] = 50:1:1:1, where M: styrene, I: initiator (ethyl-2-bromoisobutyrate), and L: ligand (*N*,*N*,*N*′,*N*″,*N*″-pentamethyldiethtylenetriamine, PMDETA). The reactants were added under N_2_. The reaction mixture was then degassed by three freeze-pump-thaw cycles and placed in a thermostatic oil bath at 85 °C. To stop the polymerization completely when this process was over, the Schlenk flask was introduced in a Dewar container filled with liquid N_2_ to freeze the reaction mixture. The mixture was then thawed to room temperature. The polymeric reaction was diluted in tetrahydrofuran (THF) and passed through a neutral alumina column to remove the copper salt. After that, the solvent was removed by evaporation and the polymers were precipitated in ethanol, filtered, washed, and dried under vacuum.

Synthesis of PS-*b*-PtBA by ATRP. The copolymer was synthesized by ATRP using the procedure above. In this case, the stoichiometry was [M]/[I]/[CuBr]/[L] = 100:1:1:1, where M: *t*-butyl acrylate, I: macroinitiator PS–Br, and L: PMDETA. The PS–Br macroinitiator was dissolved in degassed acetone (5 mL) and added to the mixture with the other reagents. Acetone enhanced the solubility of the CuBr/PMDETA complex. The reaction was carried out at 65 °C.

Hydrolysis of the PtBA Block in the PS-*b*-PAA Copolymer. The copolymers were first dissolved in CH_2_Cl_2_. Trifluoroacetic acid (TFA) was then added (10 equivalents to *t*-butyl ester units) and the mixture was stirred at room temperature for 3 days. The unprotected polymers precipitated in the reaction media and were filtered, washed with CH_2_Cl_2_, and finally dried under vacuum.

Preparation of Functionalized Polymeric Surfaces (FPSs). The copolymer PS_19_-*b*-PAA_10_ was mixed with polystyrene (20:80) and the mixture was dissolved in THF. The solution was deposited on a silicon wafer (0.5 mL, 3000 rpm, 2 min) by spin coating at 26 °C and 36% of humidity. After the deposition, the polymeric surfaces were functionalized by surface segregation of the PAA segments toward the polymer/air interface. For that purpose, the films were annealed to water vapor for 24 h at 100 °C.

Immobilization of [Ln^III^(α-SiW_11_O_39_)]^5−^ Nanoclusters on the FPSs. The [Ln^III^(α-SiW_11_O_39_)]^5−^ anions (Ln: Ce, Er) were generated in situ following Pope’s procedure for the Ce-containing POM^54^ and immobilized on the FPSs as follows:

Hybrid Surface 1 (S1). A hot (80 °C) solution of K_8_[α-SiW_11_O_39_]·13H_2_O (0.245 g, 0.076 mmol) in water (15 mL) was added dropwise to a hot (80 °C) stirred solution of CeCl_3_·7H_2_O (0.108 g, 0.290 mmol) in water (15 mL) over 8 h. The resulting yellow solution was allowed to cool to room temperature and the FPS was then immersed in the solution. After 3 days, the surface was removed from the solution, washed with abundant deionized water, and dried with an argon flow. This washing/drying process was repeated several times.

**Scheme 1:**
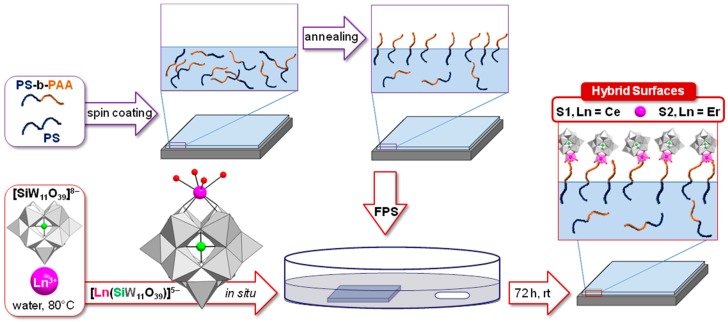
Synthetic approach followed for the preparation of functional polymeric surfaces with [Ln(H_2_O)_4_(SiW_11_O_39_)]^5−^ anions.

Hybrid Surface 2 (S2). This hybrid surface was prepared as described above but using ErCl_3_·6H_2_O (0.116 g, 0.304 mmol) as the lanthanide source. Scheme 1 shows the preparation of hybrid interfaces by immobilization of POM nanoclusters on functionalized polymeric films.

## 3. Results and Discussion

The target polymeric surfaces were made of polystyrene-*b*-poly(acrylic acid)/polystyrene blends (20:80) by spin coating on a previously cleaned silicon wafer (piranha solution at 80 °C for 30 min). The functionalization of the polymeric films was achieved by interfacial migration of the PAA block of the amphiphilic block copolymer toward the interface upon water vapor annealing. The so-generated FPSs were then immersed in aqueous POM solutions for immobilization of the inorganic nanoclusters via entrapment with the poly(acrylic acid) branches at the interface (Scheme 1).

From the vast catalog of POMs with exposed metal centers that are available in the literature, we initially focused our attention on one of the most archetypical families for testing the POM immobilization capability of the above tailored polymeric films. More specifically, we selected two members of the large family of clusters formed by a monolacunary POM fragment acting as tetradentate O-donor ligand toward a single lanthanide ion because the coordination sphere of the 4f-metal center in these anions is completed by at least four labile water molecules when present in solution. The POMs selected were two [Ln^III^(α-SiW_11_O_39_)]^5−^ anions with early (Ce) or late (Er) lanthanide ions to evaluate the influence of the 4f-metal size on the anchoring (see Scheme 1 for a polyhedral/ball-and-stick representation). These easily synthesizable and highly solution-stable species contain POM fragments derived from the well-known α-Keggin structure, which consists of a central XO_4_ tetrahedral group surrounded by four vertex-sharing M_3_O_13_ trimers, each one composed of three edge-sharing MO_6_ octahedra (X = Si^IV^ and M = W^VI^ in our case). Removal of one (M = O)^4+^ group creates a vacant site in the oxometallic shell, allowing the resulting monolacunary species to coordinate additional metallic centers (Ce or Er in our case).

**Figure 1:**
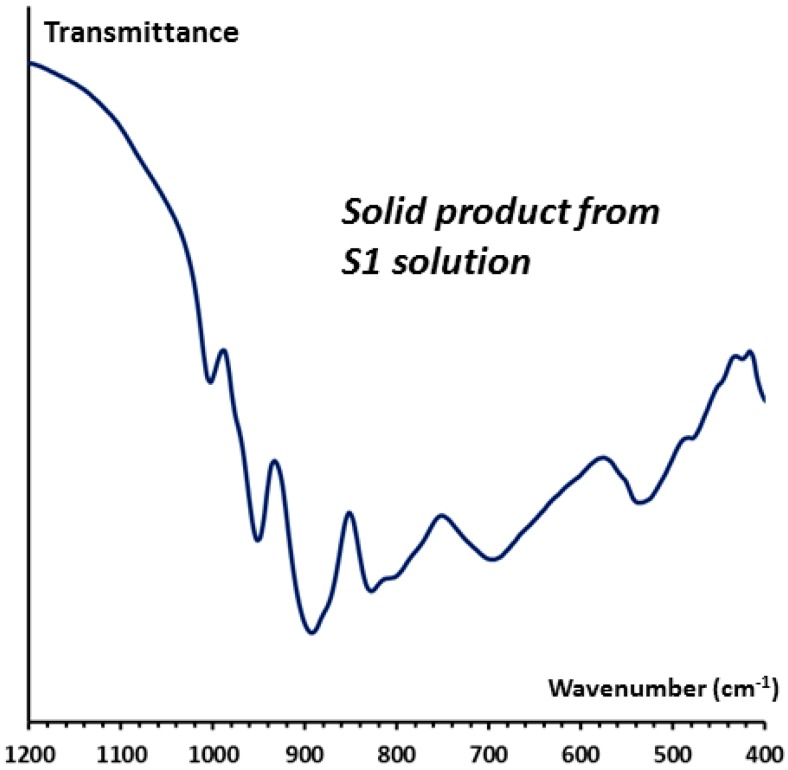
Fourier transform infrared (FT-IR) of the solid product isolated from the evaporation of S1 solution.

The selected POM nanoclusters were generated in situ by the slow addition of the preformed monolacunary [α-SiW_11_O_39_]^8−^ Keggin anion to a hot, aqueous solution containing a large excess of the corresponding lanthanide ion. According to Pope and coworkers, this large excess is required to ensure high yields of the [Ln^III^(α-SiW_11_O_39_)]^5−^ species and to avoid the formation of the related [Ln^III^(α-SiW_11_O_39_)_2_]^13−^ Peacock–Weakley sandwich-type anion as a side product.^54^ In our case, a more diluted solution was used in comparison to that employed by Pope and coworkers to avoid any kind of precipitation that could take place over the functional surface. Considering the high pH dependency of the formation of POM species in solution, the pH of both the more and less concentrated solutions were measured and proved to be virtually identical to each other (pH = 4.5). Additionally, in order to evaluate whether the dilution considerably modifies the synthetic system, the final solution of the cerium system (S1 solution) was left to evaporate in an open container until the formation of a homogeneous solid product was observed. The orange powder was identified as containing Pope’s [Ce^III^(α-SiW_11_O_39_)]_n_^5−^ anion on the basis of FT-IR spectroscopy. As shown in the IR spectrum depicted in Figure 1, both the positions and relative intensities of the vibration bands in the 400–1200 cm^−1^ POM region are in perfect agreement with those reported by Pope [FT-IR (KBr, cm^−1^): 1003 (m), 952 (s), 895 (vs), 829 (s), 802 (sh), 695 (s), 536 (m), 474 (w); where vs = very strong, s = strong, m = medium, w = weak, sh = shoulder].

After an optimum period of ca. 3 days of immersion of the FPSs in these solutions at room temperature, the resulting hybrid surfaces S1 (Ln = Ce) and S2 (Ln = Er) were gently washed with deionized water; this was to ensure the firm immobilization of the POMs on these polymeric films by getting rid of any possible cluster that was weakly grafted at the interface as a result of impregnation. The characterization of the pristine FPS and the hybrid S1 and S2 surfaces was performed using X-ray photoelectron spectroscopy (XPS) (to analyze the sample compositions), confocal Raman spectroscopy (to identify the formation of the hybrid polymer–inorganic interfaces), and atomic force microscopy (AFM) (to observe the evolution of the surface profiles). These three techniques allowed us to confirm the presence of the [Ln^III^(α-SiW_11_O_39_)]^5−^ POMs firmly immobilized on the polymeric surfaces. These results evidence the facile anchorage of POMs with exposed metal centers to the solid substrates upon design of tailored polymeric films with target coordinating functionalities on the flexible branches.

**Figure 2:**
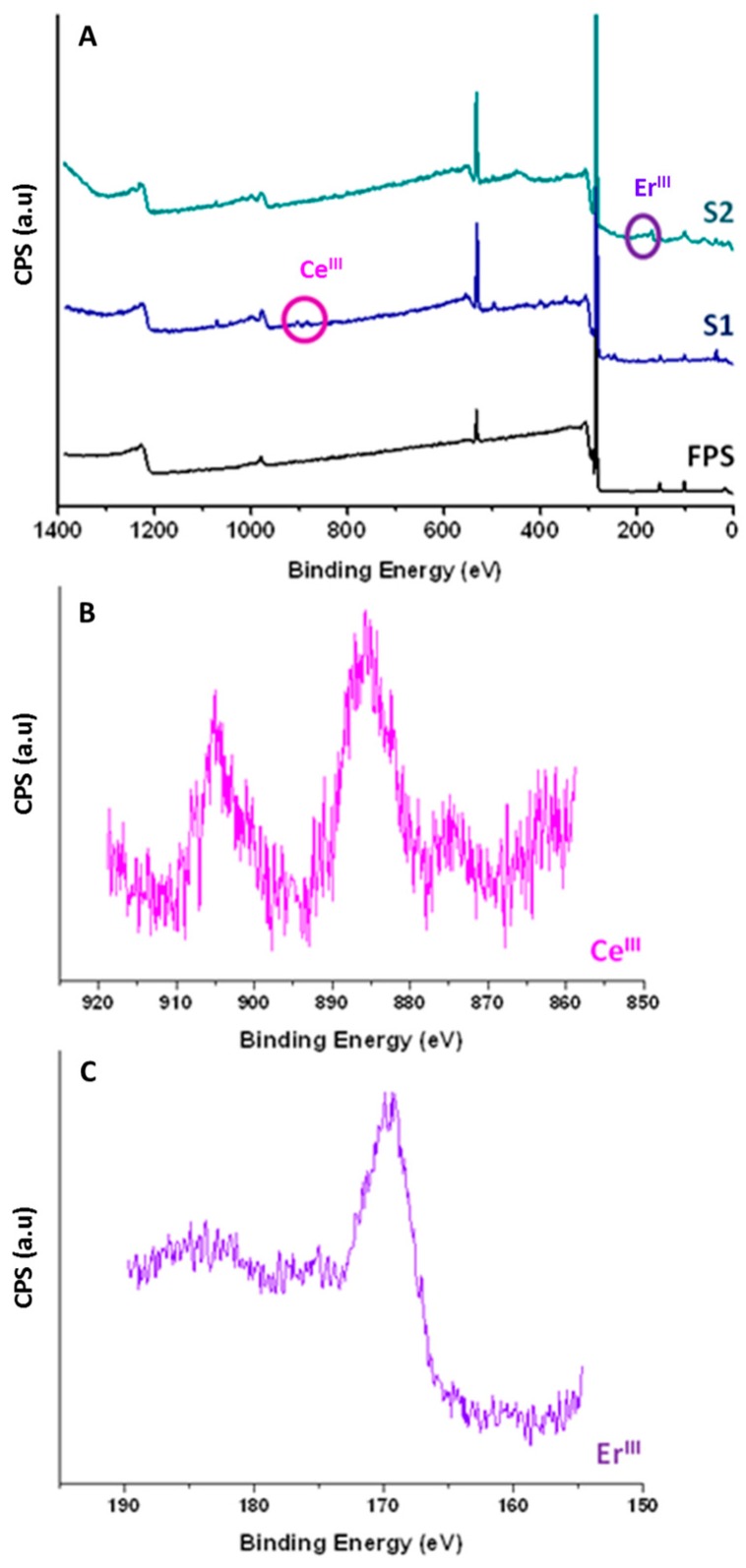
X-ray photoelectron spectroscopy (XPS) surveys of the pristine functionalized polymeric surface (FPS) and the hybrid S1 and S2 surfaces (**A**), together with details of Ce peaks in S1 (**B**) and Er peaks in S2 (**C**).

X-ray Photoelectron Spectroscopy (XPS). XPS was used to identify the elemental composition of the surfaces. Figure 2 shows the surveys of three different surfaces: the pristine FPS and the hybrid S1 and S2 (surveys with the overall compositional analysis are included in Figures S1 and S2 in the Supporting Information). The FPS survey exhibits three peaks corresponding to Si 2p (Binding energy (BE) = 102.2 eV), C 1s (BE = 285.6 eV), and O 1s (BE = 532.6 eV). The former originates from the silicon wafer whereas the two latter are indicative of the polystyrene-*b*-poly(acrylic acid) chains within the polystyrene matrix. As expected, these three peaks are retained with negligible variations in the surveys of both S1 (Si 2p, BE = 102.25 eV; C 1s, BE = 285.66 eV; O 1s, BE = 532.61 eV) and S2 (Si 2p, BE = 101.31 eV; C 1s, BE = 284.95 eV; O 1s, BE = 532.63 eV). In both cases, additional signals can be identified. For S1, two peaks corresponding to Ce 3d (BE = 904.35 eV, 884.65 eV) and the characteristic signal of W4f (BE = 36.58 eV) are observed. The survey of S2 also exhibits this W4f signal (BE = 35.56 eV) and, furthermore, one peak corresponding to Er 4d (BE = 169.54 eV).

Taking into account that the [Ln^III^(α-SiW_11_O_39_)]^5−^ anions are stable in aqueous solution as demonstrated by Pope and coworkers on the basis of ^183^W-NMR spectroscopy, the simultaneous presence of W 4f and Ce 3d/Er 4d signals in the XPS surveys of the S1 and S2 surfaces is in full agreement with the existence of lanthanide-containing monolacunary Keggin nanoclusters immobilized on the polymeric surface. Moving a step forward and taking surface S1 as a reference, the preliminary quantitative analysis was performed by the integration of the peaks and the W:Ce atom ratio was determined to be 5:1 (1.9:0.4 in absolute atom %). Since the results are far away from the theoretical ratio expected for this polyoxometalate anion (11:1), a deeper analysis of the signals belonging to Ce and W was carried out by means of high-resolution XPS.

**Figure 3:**
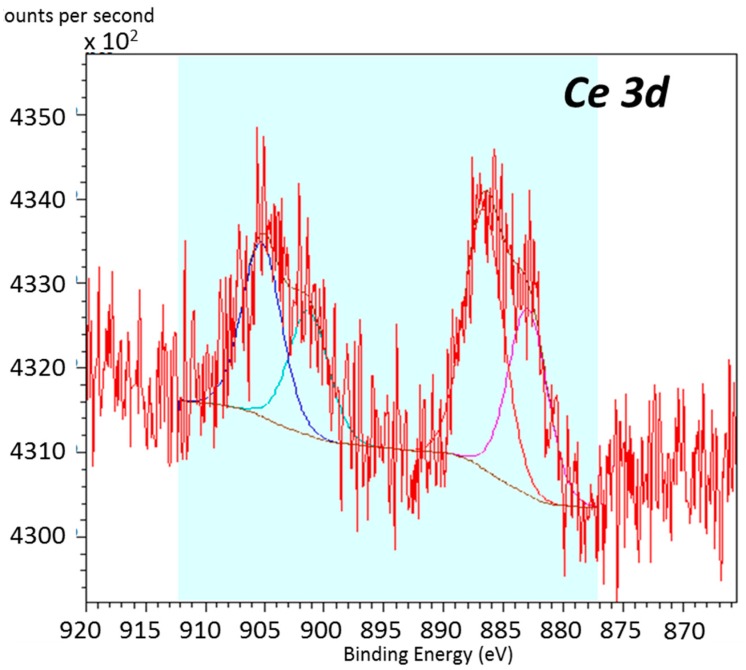
Expanded region of the Ce3d signal for the high-resolution XPS spectrum of the functionalized hybrid surface S1.

The expanded region of the Ce3d signal within the high-resolution XPS spectrum of the S1 surface is depicted in Figure 3. The fitting of the raw data indicated the presence of two different Ce species. According to the literature [65,66,67], these two species could easily correspond to O_PAA_-Ce-O_POM_ and Ce-O_PAA_ environments; that is, the first one represents the Ce^3+^ ions incorporated into monolacunary polyoxometalates, whereas the second describes the Ce^3+^ cations acting as charge compensating units due to the excess present in the reaction media and, therefore, they might be directly bonded to the carboxylic groups of the copolymer. The bond energies and atom percentages for each Ce and W moieties are compiled in Table 1. Close inspection of the data reveals that the Ce_POM_:W atom ratio calculated from the O_PAA_-Ce-O_POM_ contribution is 11:1 (1.9:0.18 in absolute atom %) in perfect agreement with the existence of [Ce^III^(α-SiW_11_O_39_)]^5−^ anions. In order to maintain the electroneutrality of the system, the calculated amount of charge compensating Ce^3+^ cations might well indicate the presence of additional K^+^ ions. From all the above, the results confirm that lanthanide-substituted Keggin-type species are covalently linked to functional polymeric surfaces.

**Figure 4:**
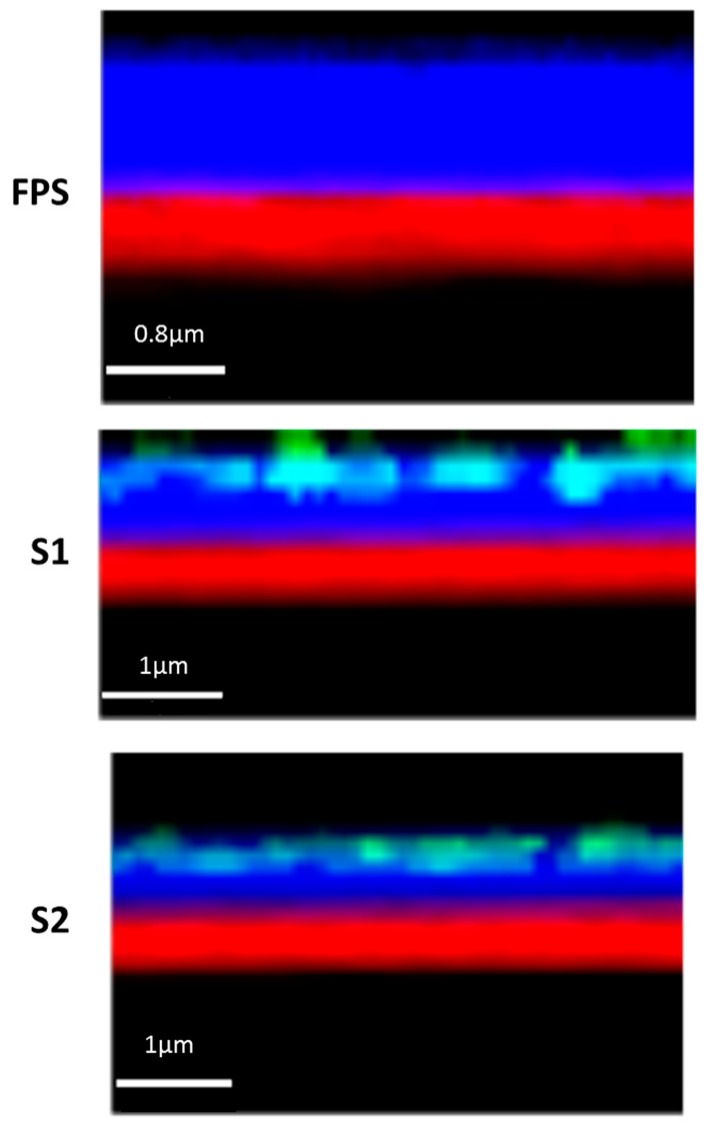
Depth profile of the surfaces before (FPS) and after the anchoring of the [Ce(α-SiW_11_O_39_)]^5−^ (S1) and [Er(α-SiW_11_O_39_)]^5−^ (S2) clusters.

Confocal Raman Spectroscopy. Straightforward immobilization of the in situ-generated [Ln^III^(α-SiW_11_O_39_)]^5−^ anions at the polymeric surface via simple immersion of the FPS-deposited wafers in aqueous POM solutions has been further confirmed by means of confocal Raman spectroscopy. Moreover, this technique also provides an interesting view about the arrangement of the nanoclusters on the surface. The signals of the three different components employed (substrate, polymeric surface, and POMs) are depicted in Figure 4 using different colors. The red color represents the silicon wafer used as substrate whereas the blue color stands for the PS-*b*-PAA/PS polymer film, which is randomly distributed within the polystyrene matrix. This is what we observed for the naked FPS. When the hybrid S1 and S2 surfaces were tested, we noticed an additional green color that covers the blue regions in a relatively homogeneous manner. This green color corresponds to the signals of Ce (228 cm^−1^) and Er (228 cm^−1^), respectively. These results clearly indicate that the selected POMs are immobilized on top of the functionalized surfaces.

**Figure 5:**
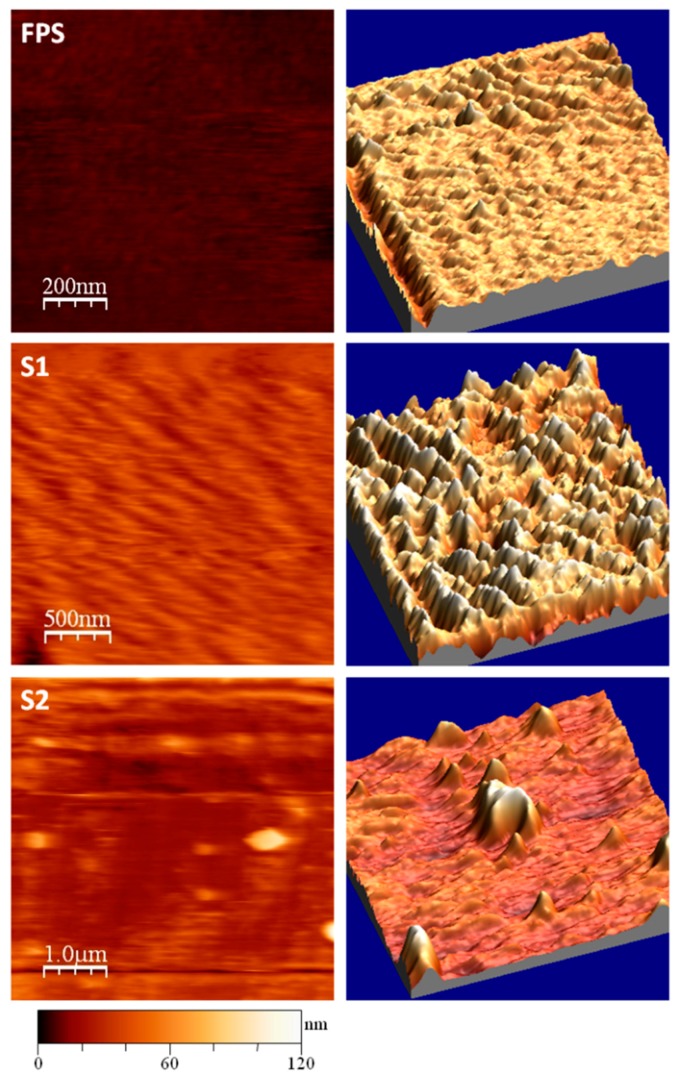
Two- and three-dimensional atomic force microscopy (AFM) images of the pristine FPS and the hybrid S1 and S2 surfaces.

Atomic Force Microscopy (AFM). Topographical studies were made using atomic force microscopy. Figure 5 displays the two- and three-dimensional images of the pristine FPS and the hybrid S1 and S2 surfaces. These images highlight the variation between the three different films. The profile of the original FPS is low and has regular peaks between 7 and 10 nm. In contrast, the S1 survey shows a relatively homogeneous distribution of peaks in the 60–70 nm range, whereas irregular peaks separated between 100 and 120 nm are observed for S2. Thus, the topographies obtained evidence not only a significant increase in the roughness of the functional polymeric films upon POM immobilization, but also that the distribution of the clusters at the interface is highly dependent on the specific nature of the exposed metal center. This is shown by the observation of closely arranged, homogeneous peaks for the large cerium ion vs. larger but irregularly distributed peaks for the smaller erbium ion. This dependence might well be indicative of the lanthanide centers being fully involved in the linkage between the coordinating PAA functionalities on flexible branches at the interface and the POM nanoclusters. However, the effect of the potential heterogeneous topographies of the parent functional surfaces cannot be disregarded [52].

## 4. Conclusions

This work describes a very facile approach to obtain hybrid polymer–inorganic surfaces by immobilization of polyoxometalate nanoclusters on tailored polymer films. We have made use of the spin coating technique to prepare polystyrene-*b*-poly(acrylic acid)/polystyrene films on silicon wafers, which have been subsequently functionalized by interfacial migration of the amphiphilic block copolymer toward the interface upon water vapor annealing. The coordinating poly(acrylic acid) functionalities at the interface have been proven to be highly effective in anchoring polyoxometalates with exposed 4f-metal centers displaying labile aqua ligands by simple immersion of the polymer films in aqueous solutions of [Ln^III^(α-SiW_11_O_39_)]^5−^ clusters generated in situ. The presence of firmly immobilized nanoclusters has been demonstrated by X-ray photoelectron and confocal Raman spectroscopies, together with atomic force microscopy.

## Figures and Tables

**Table 1:** Analysis of the Ce and W contributions: binding.

Element		Peak and BE (eV)		Atom (%)
Ce	O_PAA_-Ce-O_POM_	Ce 3d_5/2_ 883.0	Ce 3d_3/2_ 901.3	0.18
	Ce-O_PAA_	Ce 3d_5/2_ 886.7	Ce 3d_3/2_ 905.2	0.25
				Total = 0.40
W		W4f	35.6	
				Total = 1.9

## Supplementary Materials

The following are available online at http://www.mdpi.com/2079-4991/8/3/142/s1. Compositional XPS analyses for the hybrid surfaces, Figure S1: Survey and compositional XPS analysis for the hybrid S1 surface, Figure S2: Survey and compositional XPS analysis for the hybrid S2 surface.
