# Supplementary material for: Immobilization of Polyoxometalates on Tailored Polymeric Surfaces
Source: Nanomaterials (Basel). 2018 Mar 2;8(3):142. doi: 10.3390/nano8030142 (PMC5869633; doi:10.3390/nano8030142)
Supplement: Supplementary file 1 [file nanomaterials-08-00142-s001.docx]

Supporting Information

Immobilization of Polyoxometalates on Tailored Polymeric Surfaces

Saioa Aguado-Ureta ^1^, Juan Rodríguez-Hernández ^2^, Adolfo del Campo ^3^, Leyre Perez-Álvarez ^4,5^, Leire Ruiz-Rubio ^4,5^, José Luis Vilas ^4,5,^*, Beñat Artetxe ^1^, Santiago Reinoso ^1^ and
Juan M. Gutiérrez-Zorrilla ^1,5,^*


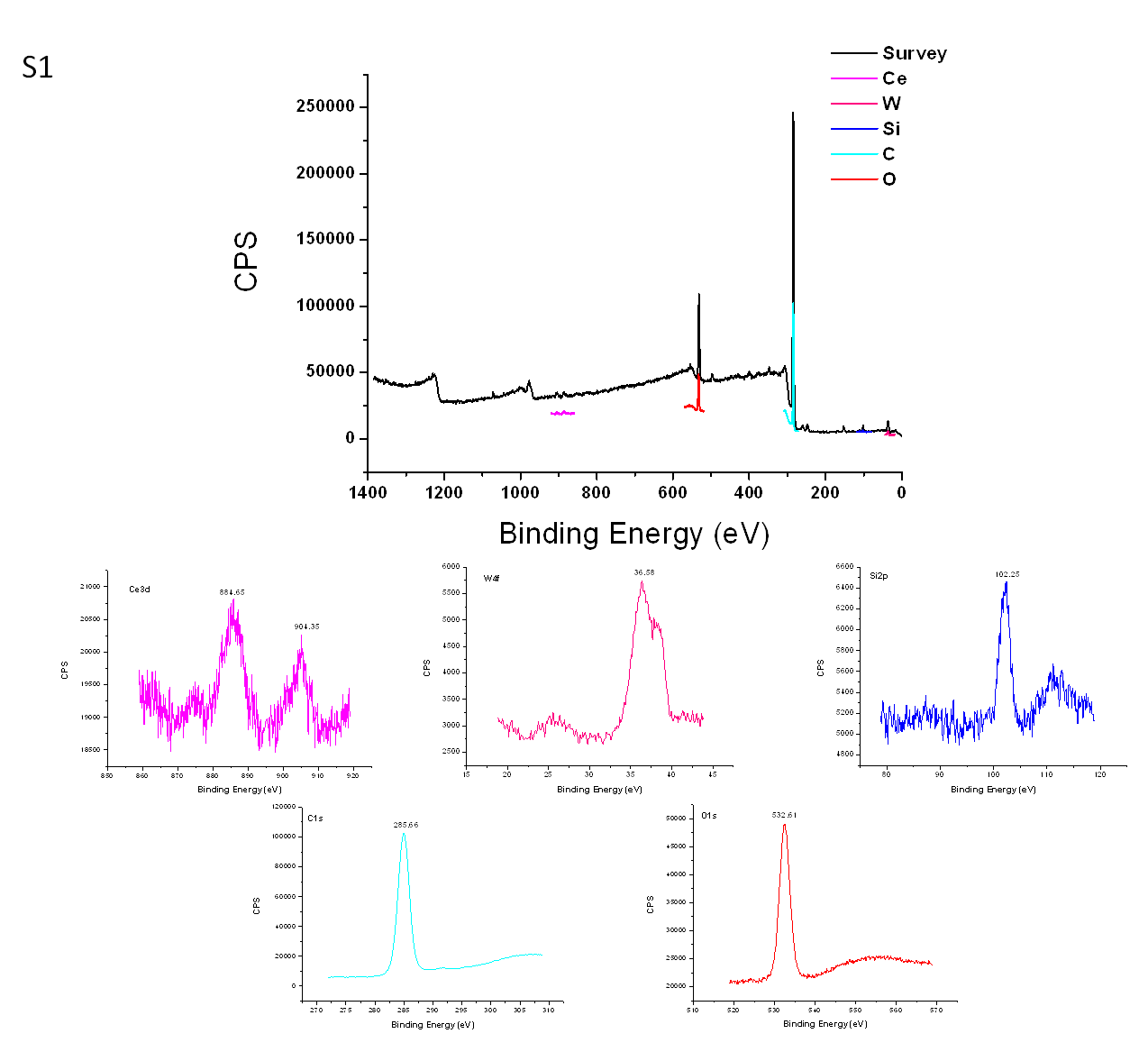


**Figure S1.** Survey and compositional XPS analysis for the hybrid S1 surface.


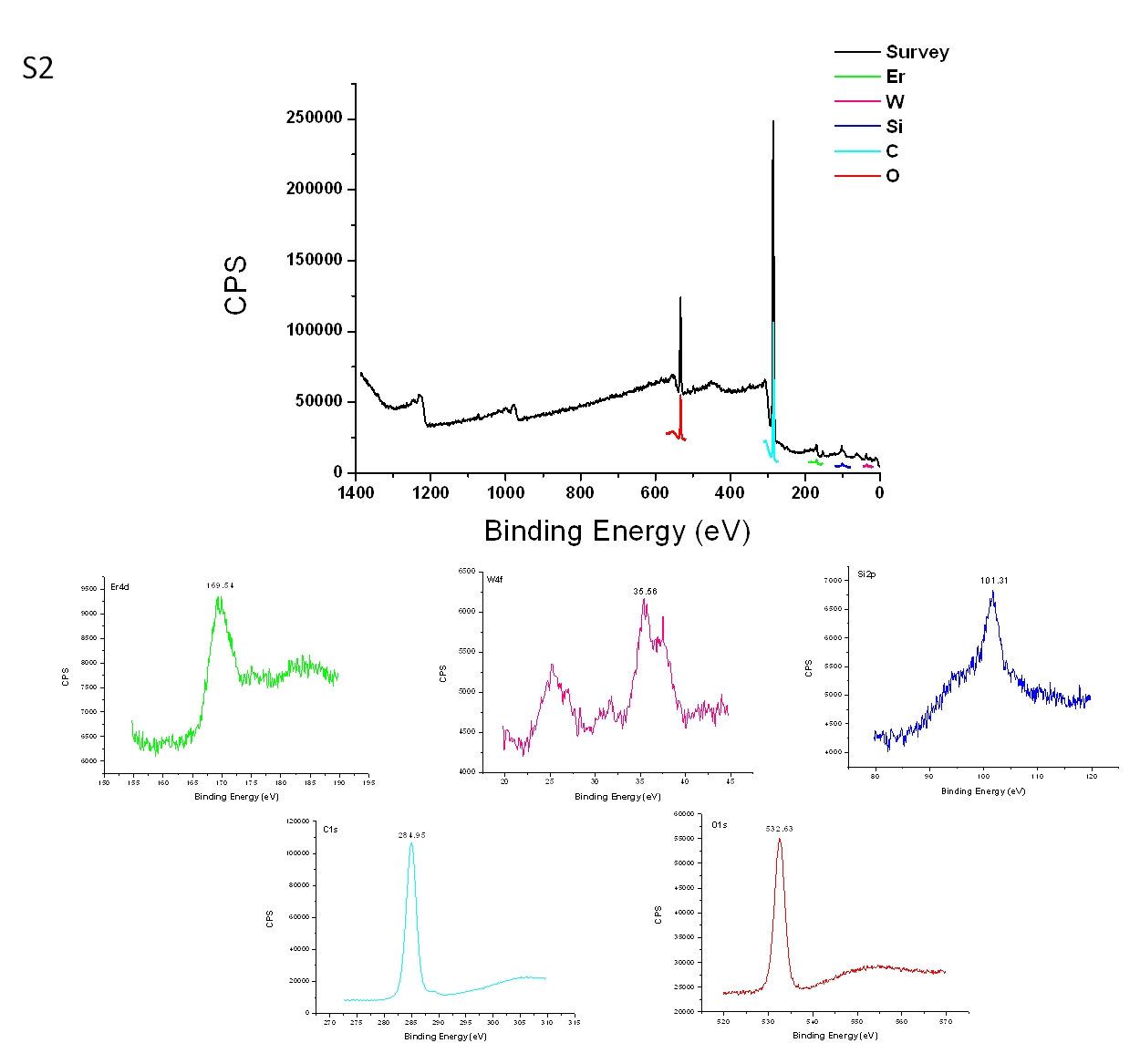


**Figure S2.** Survey and compositional XPS analysis for the hybrid S2 surface.
